# Activation of the ileal neuroendocrine tumor cell line P-STS by acetylcholine is amplified by histamine: role of H3R and H4R

**DOI:** 10.1038/s41598-017-01453-5

**Published:** 2017-05-02

**Authors:** Beatrix Pfanzagl, Diana Mechtcheriakova, Anastasia Meshcheryakova, Stephan W. Aberle, Roswitha Pfragner, Erika Jensen-Jarolim

**Affiliations:** 1 Institute of Pathophysiology and Allergy Research, Center for Pathophysiology, Infectiology and Immunology, Medical University of Vienna, Vienna, 1090 Austria; 2 The interuniversity Messerli Research Institute, University of Veterinary Medicine Vienna, Medical University Vienna, University Vienna, Vienna, 1210 Austria; 3 Department of Virology, Medical University of Vienna, Vienna, 1090 Austria; 4 Department of Pathophysiology and Immunology, Center of Molecular Medicine, Medical University of Graz, Graz, 8010 Austria

## Abstract

Neuroendocrine tumors may present with pseudoallergic reactions like diarrhea and idiopathic anaphylaxis. Here we present the P-STS human ileal neuroendocrine cell line as a model cell line for these tumors. Neuroendocrine markers and changes in cytoplasmic calcium concentration ([Ca^2+^]*i*) in response to several possible activators of 5-hydroxytryptamine (﻿5-HT) release were analyzed. P-STS cells still expressed chromogranin A and synaptophysin after 2 years of culture. Tryptophan hydroxylase 1 mRNA and a low amount of 5-HT were also detected. Acetylcholine (ACh) caused a rise in [Ca^2+^]*i*. Somatostatin inhibited, whereas histamine (HA) but not the HA receptor ligand betahistine enhanced activation by ACh. The [Ca^2+^]*i* response to ACh/HA was inhibited by the HA receptor H3 (H3R) agonist methimepip and by the antidepressant imipramine. Further [Ca^2+^]*i* response studies indicated the presence of H4Rs and of a functional calcium sensing receptor. High or low affinity IgE receptor protein or mRNA were not detected. Taken together, neuroendocrine markers and response to intestinal neurotransmitters approve the P-STS cell line as a valuable model for enterochromaffin cells. Enhancement of their ACh-induced pro-secretory response by HA, with a role for H3R and H4R, suggests an amplifying role of neuroendocrine cells in allergen-induced diarrhea or anaphylaxis.

## Introduction

Enterochromaffin (EC) cells are dispersed throughout the intestinal mucosa and produce and store the largest pool of 5-HT (serotonin) in the body. They release 5-HT from basal vesicles to the serosal side upon signals like mechanical stimulation, acidic pH, nutrients or other chemical mediators^1, 2^. Animal models indicate that the crosstalk between EC and inflammatory cells via 5-HT determines intestinal inflammation^3^. Most of these cells have apical microvilli projecting into the lumen and are supposed to function as transepithelial sensory transducers, as no nerve fibers penetrate the intestinal epithelium^4, 5^. By binding to 5-HT_4_ receptors on presynaptic membranes of afferent vagal nerve synapses of the enteric nervous system, 5-HT is thought to augment neurotransmitter release and enhance gut secretory and motility reflexes in response to natural stimuli^6–8^. Accordingly, high 5-HT levels can cause diarrhea^9^ and a role of 5-HT in the pathology of inflammatory bowel disease and other disorders of gastrointestinal motility is discussed^10, 11^.

Jejuno-ileal neuroendocrine tumors are among the most common malignant neuroendocrine neoplasms of the gastrointestinal tract^12^. Although different types of enteroendocrine cells are present in this part of the intestine^13, 14^, neuroendocrine tumors arising from the jejuno-ileum almost exclusively show EC cell differentiation^14, 15^. The cell of origin of these tumors is thought to be a committed neuroendocrine progenitor cell^14^. Ileal neuroendocrine tumors are rare, slow-growing and often only detected when they have already metastasized^16^. They can cause symptoms like diarrhea^17^, flushes, bronchoconstriction or “idiopathic” anaphylaxis^18, 19^ caused by release of biogenic amines and peptides from the tumor cells^20, 21^. These symptoms sometimes occur in response to specific foods^22^ and can be alleviated by treatment with somatostatin (SST) receptor agonists in about 70% of the patients^23^. A model cell line could be a valuable tool to study the possible context to IgE-mediated hypersensitivities.

Human cell lines of small intestinal origin represent such useful experimental models but are scarce^24^. They may upon long-term cultivation lose their neuroendocrine features (e.g. CNDT2^25^) or may be overgrown by genetically different cells present in the original culture^26^. Small numbers of Epstein Barr virus (EBV)-infected B cells transferred from the original tumor into cell culture easily outgrow slow-growing tumor cells^27^. The P-STS cell line^26, 28^, isolated from a poorly differentiated neuroendocrine tumor of the terminal ileum, grows with a stable genotype^26^. We aimed to definitely establish P-STS as a reliable 5-HT-producing EC cell line by showing stable expression of the neuroendocrine vesicle components chromogranin A (CgA) and synaptophysin and of tryptophan hydroxylase-1 (TPH1), the rate-limiting enzyme for synthesis of 5-HT expressed specifically in enteroendocrine cells^1^. Enteric 5-HT release is induced by muscarinic agonists (e.g. the endogenous agonist ACh) applied at the serosal side and involves influx of extracellular Ca^2+^ via voltage-gated L-type Ca^2+^ channels that is inhibited by SST^1, 29–31^.

In addition to these known features of EC cells, we investigated the response of P-STS cells to other intestinal neurotransmitters (the β-adrenergic agonist isoproterenol, γ-aminobutyric acid (GABA) and 5-HT) and to histamine (HA), a consumed or endogenously generated molecule implicated in food intolerance and allergic reactions. We also screened for the presence of IgE receptors that might contribute to diarrhea, flushes or anaphylaxis associated with neuroendocrine tumors via immunoglobulin-mediated mechanisms of vesicle release. As a further step of characterization we investigated whether a [Ca^2+^]*i* rise is evoked by ligands of the calcium sensing receptor (CaSR) which plays an important role in intestinal secretion and nutrient sensing^32–34^.

## Results

### P-STS cells express neuroendocrine markers and are free of EBV

**Figure 1:**
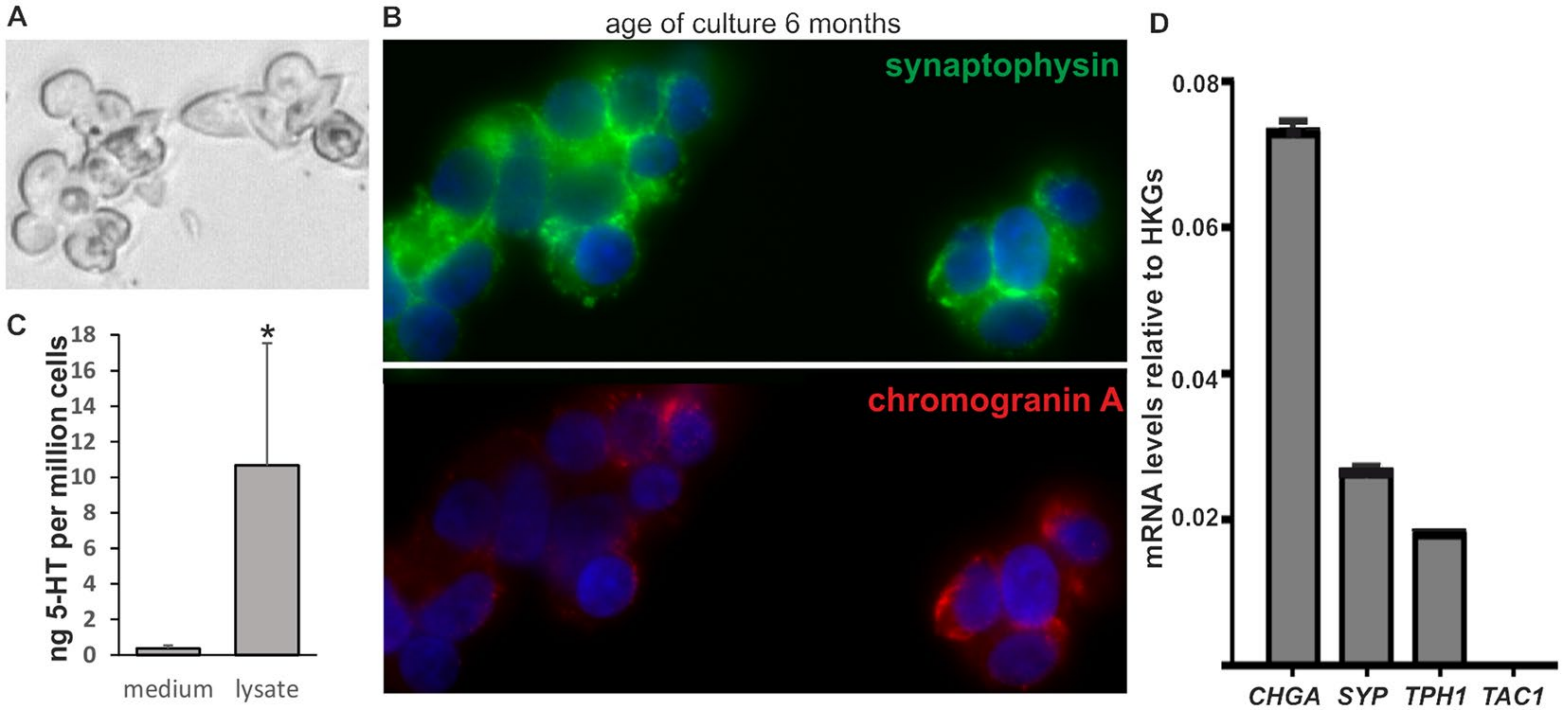
Expression of neuroendocrine markers in P-STS cells. (**A**) Light microscopy picture of P-STS cells adhering to the culture dish, (**B**) Immunofluorescence staining with anti-CgA (Cy3-labeled secondary antibody) and anti-synaptophysin (Alexa 488-labeled secondary antibody) after 6 months of continuous cultivation, (**C**) 5-HT in the supernatant after 1 h of incubation of adherent P-STS cells (continuously cultivated for 10 months) in serum-free medium containing 5% BSA at room temperature and in the cell lysate as determined by ELISA. *p < 0.05 as calculated by the unpaired two-tailed Student’s *t*-test assuming equal variances. (**D**) Expression of neuroendocrine marker genes in P-STS cells assessed by real-time PCR analysis. Real-time PCR analysis was performed using the ΔCt method for relative quantification. Expression mRNA levels of the target gene were normalized to the average of the HKGs. Shown are genes encoding neuroendocrine markers such as *CHGA* (encoding chromogranin A), *SYP* (encoding synaptophysin), *TPH1* (encoding tryptophan hydroxylase 1), and *TAC1* (encoding the tachykinin peptide hormone family, substance P and neurokinin A, as well as the related peptides). Results are shown as mean values in one experiment ± SD and are representative of two independent experiments.

P-STS cells were growing semi-adherently (Fig. 1A) with a doubling time of about one week. Immunofluorescence staining showed expression of CgA and synaptophysin as expected for neuroendocrine cells^35^ after 6 months of continuous cultivation (Fig. 1B). In the cell lysate of P-STS cells 5-HT (10.7 ± 6.8 ng per 10^6^ cells or 41 ± 26 ng mg^−1^ cell protein) was detected by ELISA (Fig. 1C). In comparison, the 5-HT content of native human ileal EC cells after several purification steps certainly causing 5-HT release from these touch-sensitive cells was 180 ng/mg cell protein^36^. Real-time PCR analysis confirmed the expression of CgA, synaptophysin and TPH1, the rate-limiting enzyme for 5-HT synthesis, on the mRNA level, while *TAC1*, another neuroendocrine marker mRNA, was not detected (Fig. 1D). Importantly, no EBV nucleic acids were detected by real-time PCR in about 10^5^ P-STS cells, while in accordance with their known status of infection according to ATCC, in a similar number of Raji B cells 1.2 × 10^6^ copies of EBV nucleic acid were detected. This shows that the P-STS neuroendocrine cells have not been overgrown by co-isolated EBV-infected lymphocytes during cell line establishment.

### P-STS cells show a [Ca^2+^]*i* response to ACh and CaSR agonists

**Figure 2:**
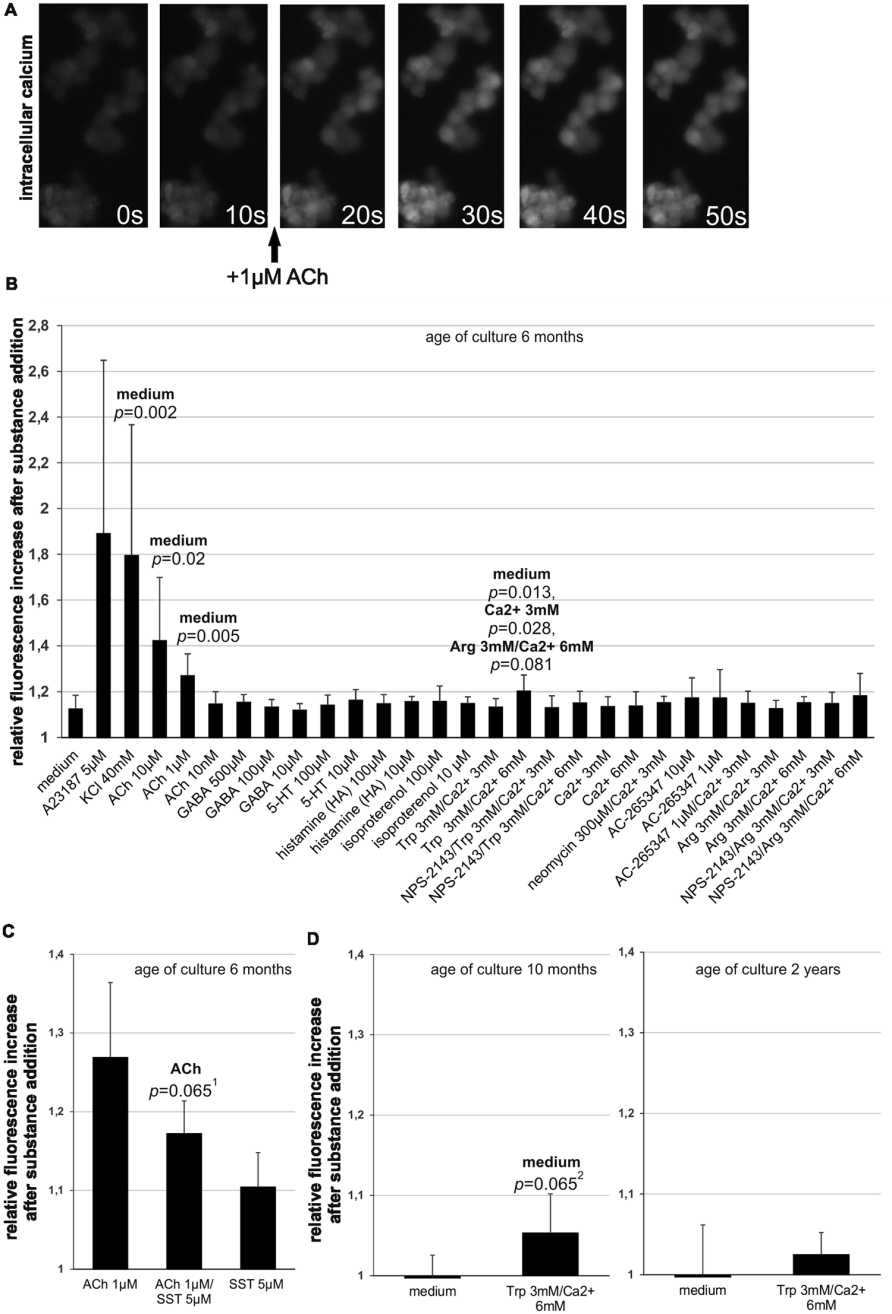
Intracellular [Ca^2+^]*i* response of P-STS to addition of neurotransmitters and CaSR ligands. (**A**) Time course of intracellular fluorescence of a group of Fluo-4 AM-stained P-STS cells after treatment with 1 µM ACh. (**B**) Screening experiment (see explanation in *Methods*) for fluorescence enhancement measured 10 s after the start of substance addition in relation to baseline fluorescence. Final substance concentrations are indicated. NPS-2143 (final concentration 2 µM) was added 1 min before taking the first time point for baseline determination. Mean values and standard deviations were calculated from 3–15 independent samples. Statistical significance compared to the treatment group indicated on top of the bar was calculated by univariate analysis with the Mann-Whitney *U*-test. (**C**) Inhibition of ACh-induced P-STS activation by somatostatin. Mean values and standard deviations were calculated from 6 experiments. ^1^ *p* = 0.059 when calculated from ranked data by the unpaired two-tailed *t*-test assuming unequal variances. (**D**) [Ca^2+^]*i* response to 3 mM Trp/6 mM Ca^2+^ after 10 months and 2 years of continuous cultivation. Mean values and standard deviations were calculated from 6 experiments. ^2^ *p* = 0.049 when calculated from ranked data by the unpaired two-tailed *t*-test assuming unequal variances.

**Figure 3:**
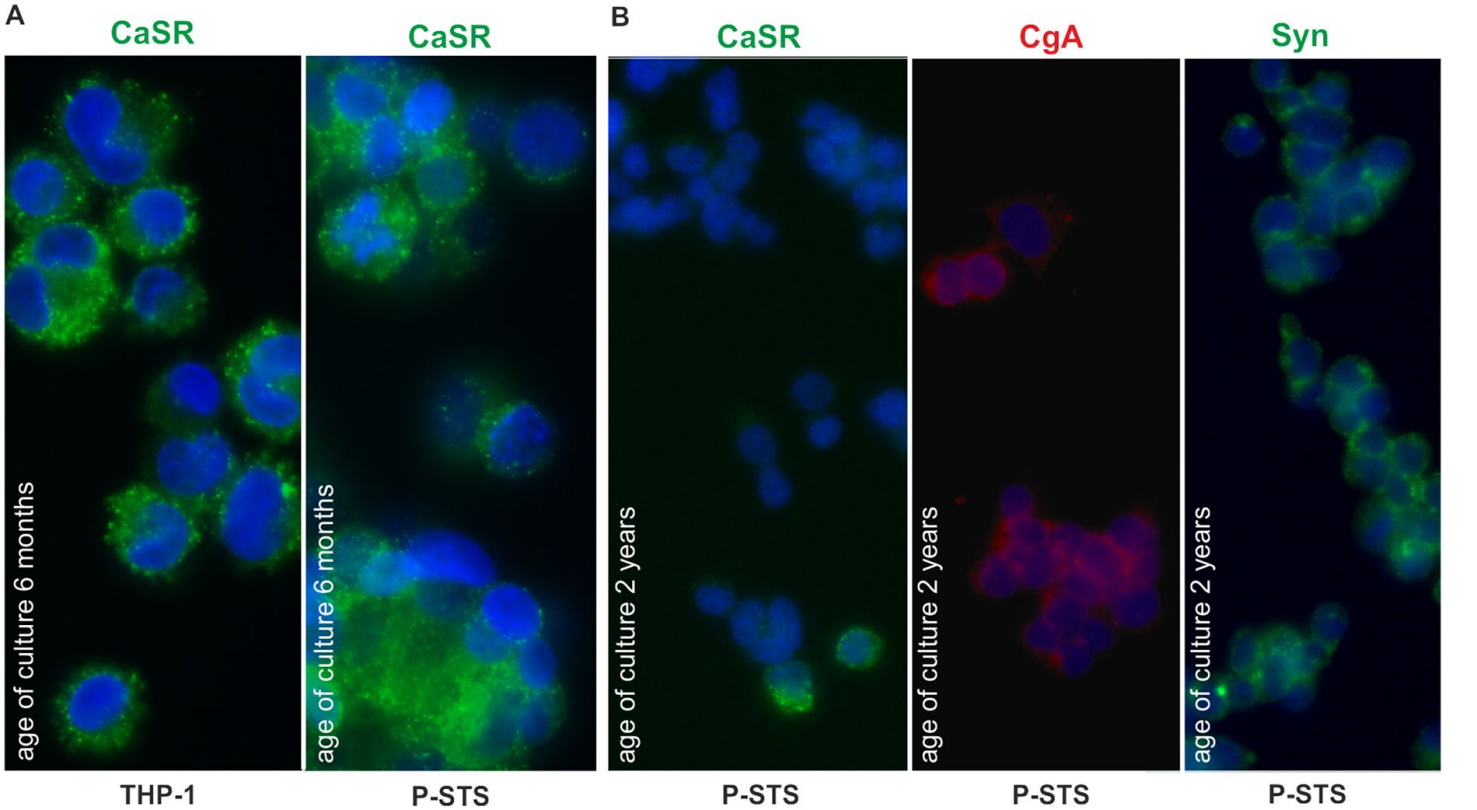
Expression of CaSR by P-STS cells. (**A**) Immunofluorescence staining with anti-CASR (Alexa 488-labeled secondary antibody) of THP-1 cells grown with 200 nM PMA for 2 d (control) and P-STS cells after 6 months of continuous cultivation. (**B**) Immunofluorescence staining with anti-CASR (Alexa 488-labeled secondary antibody), anti-CgA (Cy3-labeled secondary antibody) and anti-synaptophysin (Alexa 488-labeled secondary antibody) of P-STS cells after 2 years of continuous cultivation.

P-STS cells were further characterized by their reaction to putative agonists of vesicle release. First it was established that the Ca^2+^ ionophore A23187 as well as membrane depolarization by 40 mM KCl evoked an immediate rise in [Ca^2+^]*i* (Fig. 2B). Then the effect of several intestinal neurotransmitters or related substances (ACh, the β-adrenergic activator isoproterenol, GABA, 5-HT, and HA^37^) and of CaSR agonists (Ca^2+^, tryptophan (Trp) combined with Ca^2+^, neomycin and the calcimimetic AC-265347)^34, 38^ on [Ca^2+^]*i* was evaluated in a screening experiment. Of all neurotransmitters tested, only ACh induced a rise in [Ca^2+^]*i* significantly exceeding that induced by addition of pure medium alone (Fig. 2A and B). As expected, the [Ca^2+^]*i* rise induced by ACh was inhibited by SST (*p* ≤ 0.065, Fig. 2C). Despite the strong activating effect of 10 µM ACh, neither 5-HT nor CgA were detectable by ELISA in the supernatants of cells grown to final density in 24-well dishes after treatment with this neurotransmitter (data not shown). This is in accordance with the low 5-HT content of P-STS cells. It has been observed previously that treatment of rabbit duodenal tissue with ACh releases only about 2% total tissue 5-HT assumed to be contained in EC cells^30^. In our experiments this amounts to a release of about 0.2 ng 5-HT per 10^6^ cells. With a final cell density of 0.6 × 10^6^ cells per well in 200 µl of medium the 5-HT concentration achieved following release of 2% of total 5-HT would correspond to the detection level of the ELISA. Among the CaSR receptor ligands, AC-265347 appeared to have some enhancing effect on [Ca^2+^]*i*, but only Trp in the presence of 6 mM Ca^2+^ provoked a rise in [Ca^2+^]*i* significantly higher than medium alone or 3 mM Ca^2+^ (Fig. 2B). In the presence of NPS-2143, an inhibitor of the CaSR^39^, this activating effect was lost. In contrast to Trp which in the presence of Ca^2+^ is an allosteric CaSR activator, arginine (Arg) is a Ca^2+^-dependent agonist of GPRC6A, another G-protein coupled receptor that might be present on EC cells^40^, but no CASR ligand. Substitution of Trp with Arg in the presence of 6 mM Ca^2+^ did not cause a significant rise in [Ca^2+^]*i*, however. As the reaction of the cells to Trp in the presence of 6 mM Ca^2+^ was weak, we retested the cells in a separate experiment which confirmed the presence of functional CaSRs (*p* ≤ 0.065, Fig. 2D, left panel). Upon further continuous cultivation the generation time of the cells became shorter (2–3 days) and cells cultivated for 2 years had largely lost their reaction to Trp in the presence of 6 mM Ca^2+^ (Fig. 2D, right panel). Immunofluorescence staining of P-STS cells for CaSR was positive in cells cultivated for 6 months and comparable to THP-1 cells used as staining control^41^ (Fig. 3A). In accordance with the functional assay CaSR staining was lost in almost all cells after 2 years of continuous cultivation while CgA and synaptophysin were still present (Fig. 3B).

### HA enhances the [Ca^2+^]*i* response to Ach

**Figure 4:**
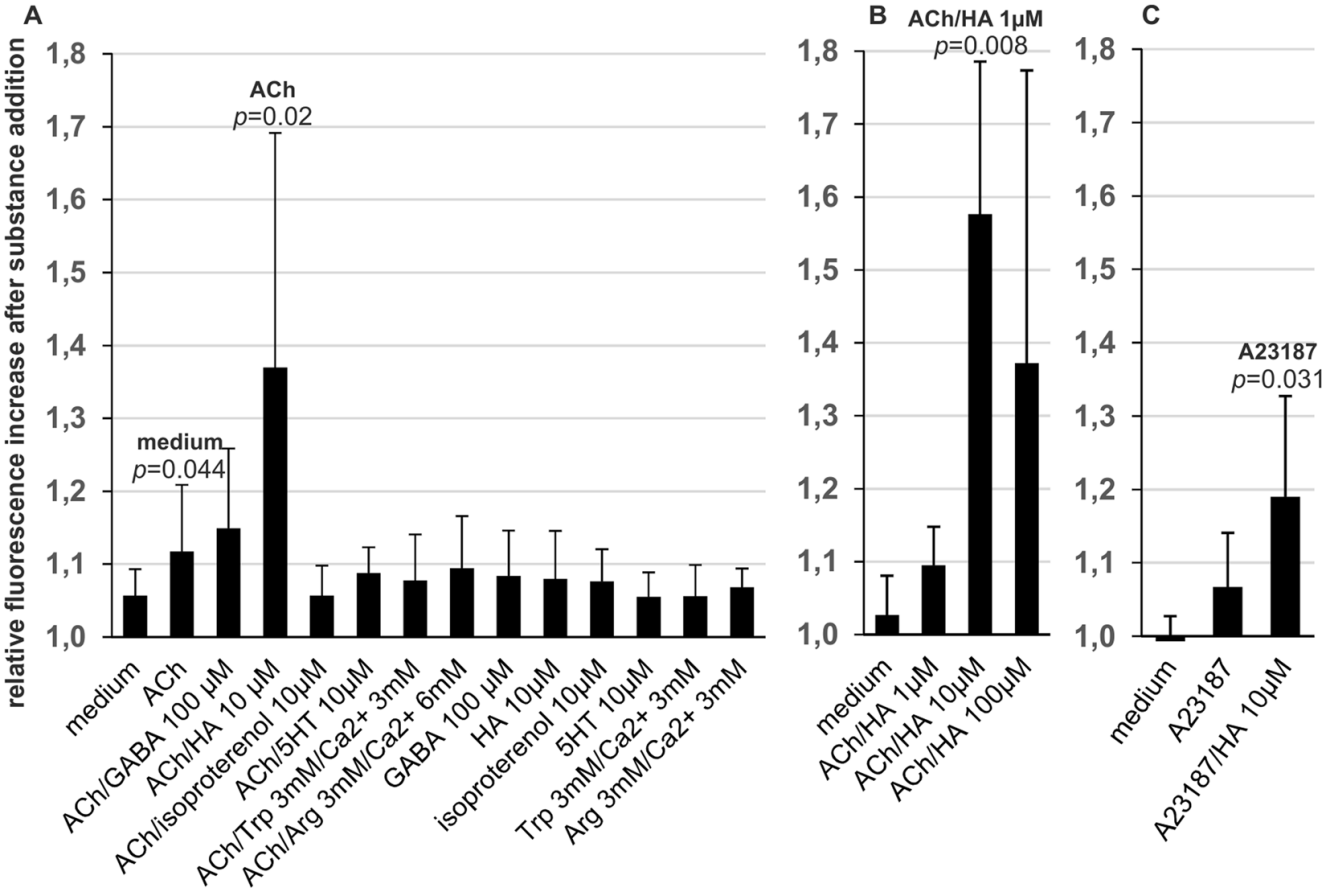
Screening for putative activators or inhibitors of the [Ca^2+^]*i* response to ACh and the Ca^2+^ ionophor A23187. Fluorescence enhancement was measured 10 s after addition of 200 µl medium containing ACh or A23187 (final concentrations 1 and 0.1 µM, respectively) and the putative activating/inhibiting substance in relation to baseline fluorescence. Final substance concentrations are indicated. Statistical significance compared to the treatment group indicated on top of the bar was calculated by univariate analysis with the Mann-Whitney *U*-test. (**A**) Screening test (see explanation in *Methods*) for P-STS activation by putative activators/inhibitors of [Ca^2+^]*i* response to ACh (age of culture 10 months). Mean values and standard deviations were calculated from 6–14 independent samples. (**B**) Dependence of the [Ca^2+^]*i* response to ACh on HA concentration (age of culture > 1 year). Mean values and standard deviations were calculated from 5 experiments testing. (**C**) Influence of 10 µM HA on the [Ca^2+^]*i* rise caused by A23187 (age of culture > 1 year). Mean values and standard deviations were calculated from 9 experiments.

In a second set of [Ca^2+^]*i* response experiments compounds not eliciting a significant response when added to P-STS cells were screened for their capability to enhance or inhibit the response to 1 µM ACh (Fig. 4A). Again 100 µM GABA, 10 µM 5-HT, 10 µM of the β-adrenergic agonist isoproterenol and Trp or Arg in the presence of 3 mM Ca^2+^ were without significant effect compared to medium alone. When added simultaneously with ACh, 10 µM isoproterenol appeared to have an inhibitory effect on the [Ca^2+^]*i* response evoked by ACh (*p* = 0.106) in this screening experiment. This effect was, however, not reproducible when the cells were tested again (not shown). HA added together with ACh significantly enhanced the [Ca^2+^]*i* response elicited by medium to which only ACh was added. Further experiments showed that lowering the HA concentration from 10 to 1 µM abolished this enhancing effect, while an increase to 100 µM did not result in further acceleration of the [Ca^2+^]*i* response compared to 10 µM HA (Fig. 4B).

### HA causes a strong [Ca^2+^]*i* response in the presence of the Ca^2+^ ionophore A23187

As a first step to elucidate the mechanism behind the apparent synergism between ACh and HA, ACh was substituted by the Ca^2+^ ionophore A23187. An amount of A23187 causing only a slight increase in [Ca^2+^]*i* when added alone was sufficient to generate a strong [Ca^2+^]*i* response in the presence of 10 µM HA (Fig. 4C). This suggests that the primary contribution of ACh to the observed effect might be to cause an increase in [Ca^2+^]*i*.

### Effect of HA receptor antagonists

**Figure 5:**
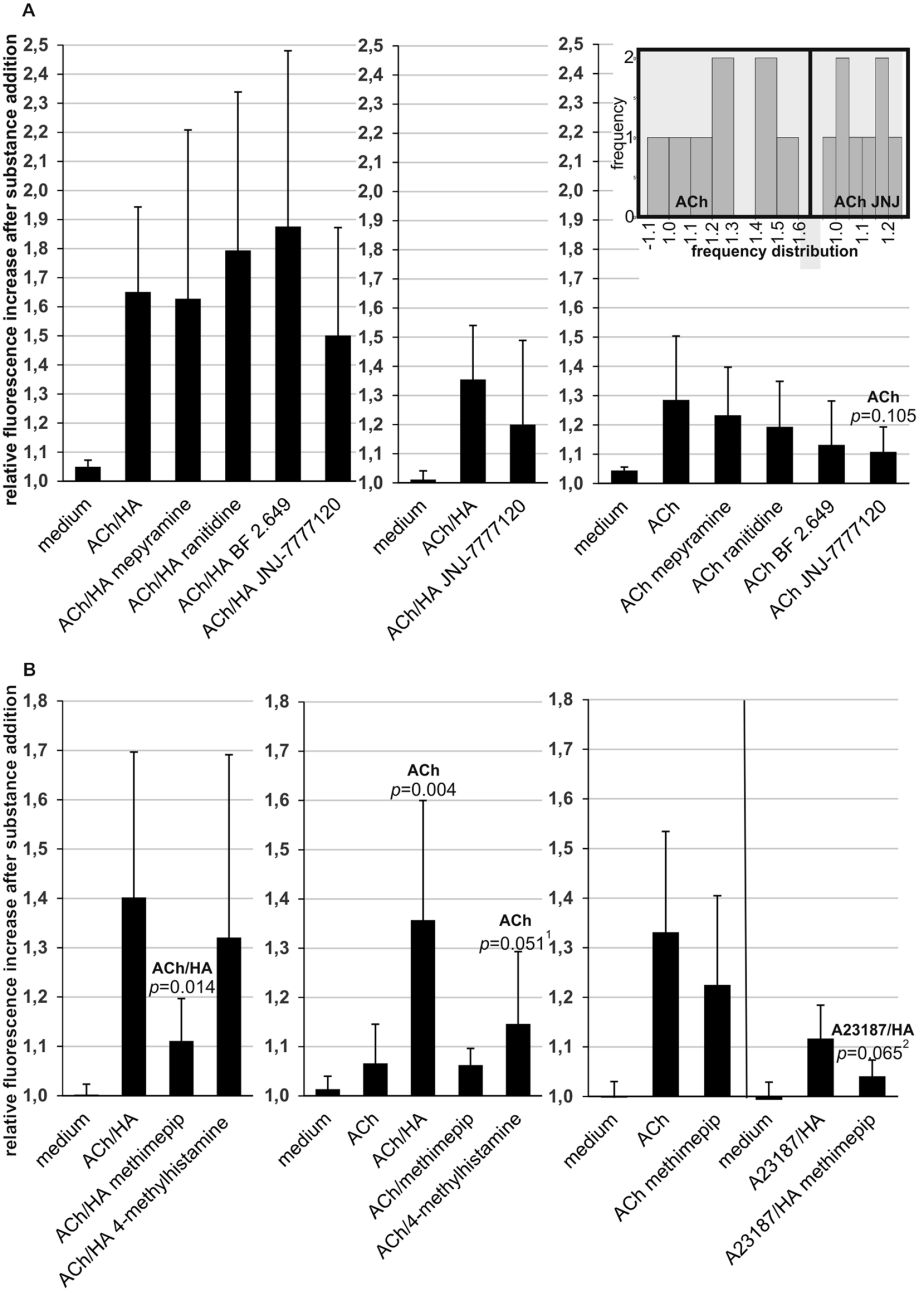
Effects of HA antagonists/inverse agonists and HA agonists on the [Ca^2+^]*i* response to ACh/HA, ACh or A23187/HA (age of culture > 1 year). Fluorescence enhancement was measured 10 s after addition of 200 µl medium containing ACh or A23187 (final concentrations as described below) with or without HA, methimepip, 4-methylhistamine or JNJ-7777120 (all added to final concentrations of 10 µM if not indicated otherwise). For preincubations with HA antagonists/inverse agonists the indicated substances or medium alone were added in 50 µl volume 2 min before taking the first time point for determination of baseline fluorescence, e.g. 10 s before addition of ACh or ACh/HA. In this case mepyramine, BF2.649, JNJ-7777120 were added to final concentrations of 1 µM and ranitidine to a final concentration of 10 µM. Statistical significance compared to the treatment group indicated on top of the bar was calculated by univariate analysis with the Mann-Whitney *U*-test. Mean values and standard deviations were calculated from at least 4 experiments. (**A**) Left panel: Inhibition of the [Ca^2+^]*i* response to ACh/HA (final concentrations 1 and 10 µM, respectively) by preincubation with HA receptor antagonists/inverse agonists (6 experiments), middle panel: effect of a higher concentration (10 µM) of JNJ-7777120 simultaneously added with ACh/HA (concentrations as in left panel, 6 experiments), right panel: inverse agonist effects of preincubation with HA receptor antagonists/inverse agonists on the [Ca^2+^]*i* response evoked by ACh (final concentration 2 µM, 8 experiments). The inset calculated with IBM SPSS Statistics shows the frequency distribution of the magnitude of [Ca^2+^]*i* response to treatment with ACh with and without preincubation with JNJ-7777120 derived from the same data. (**B**) Left panel: effect of methimepip and 4-methylhistamine on the [Ca^2+^]*i* response evoked by ACh/HA (final concentrations 1 and 10 µM, respectively, 4 experiments), middle panel: effect of methimepip and 4-methylhistamine on the [Ca^2+^]*i* response to ACh (final concentration 1 µM, 6 experiments), right panel: effect of methimepip on the [Ca^2+^]*i* response to ACh (final concentration 2 µM, 9 experiments) and A23187/HA (final concentrations 0.1 and 10 µM, respectively, 6 experiments). ^1,2^ *p* = 0.035 (^1^) and *p* = 0.049 (^2^) when calculated from ranked data by the unpaired two-tailed *t*-test assuming unequal variances.

Preincubation of the cells with selective HA receptor antagonists^42, 43^ before simultaneous addition of 1 µM ACh and 10 µM HA (ACh/HA) had little effect on the [Ca^2+^]*i* response (Fig. 5A, left panel). In accordance with published receptor affinities and inhibitory/effective concentrations, the H1R antagonist mepyramine with a pK_i_ of ~8.8^43^ at the H1R (as compared to the HA pEC_50_ of ~7^42^) and the H2R antagonist ranitidine with a pKi of 6.7 at the H2R (while the pEC_50_ of HA is 5.8^43^) were added at concentrations of 1 and 10 µM, respectively. Considering their receptor binding affinities^42, 43^ these concentrations were supposed to substantially inhibit the effect of 10 µM HA without inhibiting ACh receptors or ion channels^42^. BF2.649 has an almost 100-fold higher affinity to the human H3R than HA^44^ and a concentration of 1 µM was supposed to antagonize the effect of 10 µM HA at a putative H3R without causing unwanted side effects. The lack of inhibitory effects of H1R, H2R as well as H3R antagonists suggests that neither of these receptors is significantly involved in the enhancing effect of HA. Concerning the H4R, the binding affinities of HA and the specific antagonist JNJ-7777120^42, 45^ are similar. 1 µM of this antagonist nevertheless was previously able to inhibit 10 µM HA by 50% in a functional assay^45^. No significant inhibition was observed in our experiment (Fig. 5A, left panel). Likewise, addition of 10 µM JNJ-7777120 simultaneously with 10 µM HA (e.g. without preincubation) did not have any significant effect on the [Ca^2+^]*i* response to ACh/HA (Fig. 5A, middle panel).

### Testing of inverse agonist activities of HA receptor antagonists indicates H4R activity in P-STS cells

For all antagonists used in this work inverse agonist activity, e.g. an inhibitory effect on a putative constitutive receptor activity in the absence of HA, has been observed^43, 46–48^. Therefore the effect of preincubation with these substances on the [Ca^2+^]*i* response to 2 µM ACh in the absence of HA was tested (Fig. 5A, right panel). Again, mepyramine and ranitidine were without significant effect giving no indication for the presence of functional H1R or H2R receptors. The H3R inverse agonist/antagonist BF 2649 appeared to be inhibitory, but no significance was obtained in 8 experiments. JNJ-7777120 at a concentration of 1 µM was the only inverse agonist/antagonist with an effect on the ACh-evoked [Ca^2+^]*i* response that came near to reaching statistical significance (*p* = 0.105). The inset in Fig. 5A illustrates that the extended apparently bimodal frequency distribution of the magnitude of the [Ca^2+^]*i* response obtained with ACh substantially narrows after preincubation with JNJ-7777120. At a concentration of 1 µM side effects of JNJ-7777120 on other cellular components are unlikely^45^. Consequently the inhibition of the ACh-evoked [Ca^2+^]*i* response by this low concentration of JNJ-7777120 indicates that P-STS cells express functional H4Rs. It furthermore suggests that H4Rs positively contribute to the cellular response to ACh by displaying constitutive activity. Inhibition of this constitutive activity by an H4R inverse agonist/antagonist reducing the responsiveness to ACh may explain the effect observed with JNJ-7777120.

### Effect of H3R and H4R agonists

H3R agonists inhibit the release of neurotransmitters in the nervous system^46^. In P-STS cells, the selective H3R agonist methimepip^43, 49^ (10 µM) strongly inhibited the [Ca^2+^]*i* response to ACh/HA when added simultaneously (Fig. 5B, left panel). As methimepip was unable to significantly reduce the [Ca^2+^]*i* response to ACh in the absence of HA (Fig. 5B, middle panel), it appears unlikely that the observed inhibitiory effect on the [Ca^2+^]*i* response to ACh/HA was due to interaction of methimepip with the ACh receptor. Furthermore, in accordance with competition at the H3R, methimepip also inhibited the rise in [Ca^2+^]*i* caused by HA in the presence of the Ca^2+^ ionophore A23187 (*p* ≤ 0.065, Fig. 5B, right panel).

The selective H4R agonist 4-methylhistamine^43^ had a significant enhancing effect on the [Ca^2+^]*i* response to ACh (*p* ≤ 0.051, Fig. 5B, middle panel). Despite similar binding affinity to H4R^43^ 4-methylhistamine had a weaker enhancing effect than seen with equimolar HA in our experiment. Nevertheless, our observations denote H4R as a candidate for mediating the enhancement of the cellular response to ACh by HA, as there is no strict correlation between binding affinities of HA receptor agonists or antagonists and activities in functional assays^50^.

### Effect of the antipsychotic betahistine and the tricyclic antidepressant imipramine

**Figure 6:**
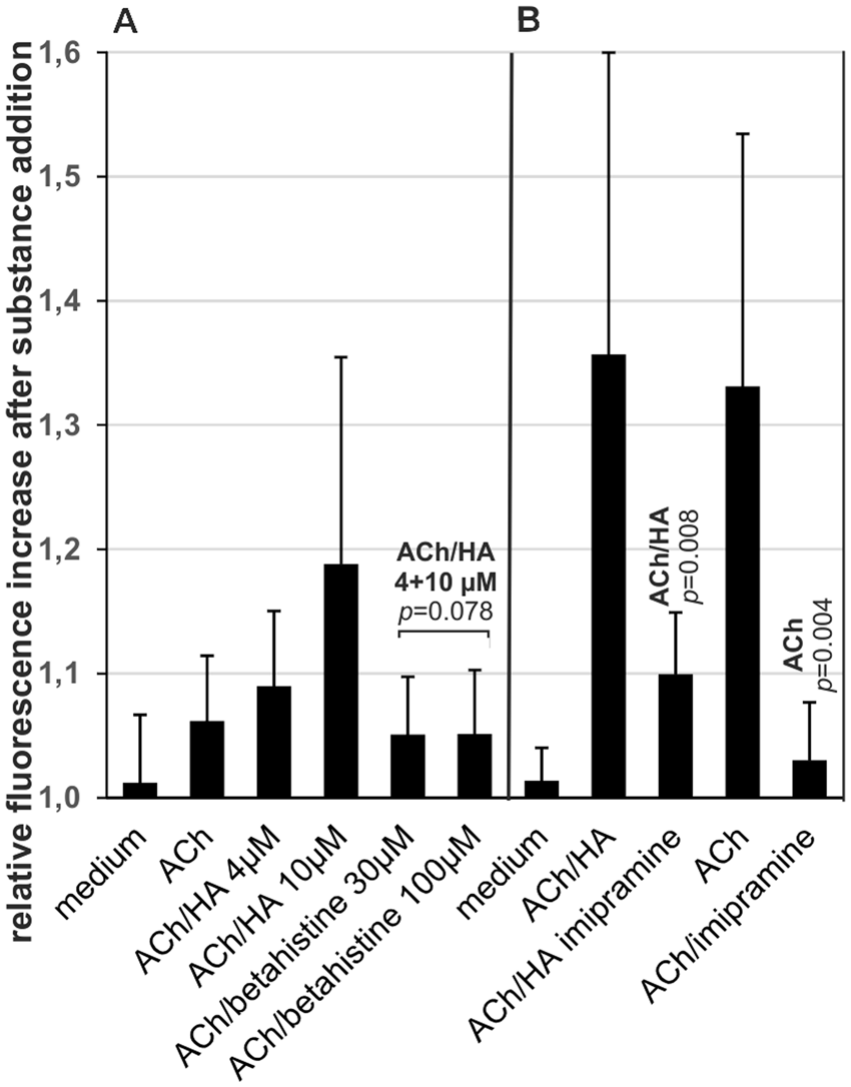
Effect of betahistine and imipramine on the [Ca^2+^]*i* response evoked by ACh/HA or ACh alone (age of culture > 1 year). Fluorescence enhancement was measured 10 s after addition of 200 µl medium containing ACh or ACh/HA and the indicated substances. Statistical significance compared to the treatment group indicated on top of the bar was calculated by univariate analysis with the Mann-Whitney *U*-test. Mean values and standard deviations were calculated from at least 6 experiments. (**A**) Comparison of the effects of betahistine and HA. Betahistine or HA were added to the cells simultaneously with ACh (final concentration 1 µM, 6 experiments) at the indicated final concentrations. As indicated by the bracket, in the Students’s t-test all betahistine samples, irrespective of betahistine concentration, were compared to the samples with HA added to a final concentration of 10 µM. (**B**) Effect of imipramine. Imipramine was added simultaneously with ACh/HA (final concentrations 1 and 10 µM, respectively, 6 experiments) or with ACh (final concentration 2 µM, 7 experiments) as indicated. Note that ACh in the absence of HA was used at a concentration of 2 µM to attain a similar response as in the presence of HA for better comparability of the inhibitory capacity of imipramine.

Betahistine, a H1R agonist, has a pEC_50_ at H1R of ~6, e.g. about ten times lower than HA^42^. It did not enhance the [Ca^2+^]*i* response to 1 µM ACh at concentrations about three and ten times higher than HA in our experiments (e.g. 30 and 100 µM, Fig. 6A). This again indicates that H1R does not play an important role in the [Ca^2+^]*i* response to ACh. Imipramine, another psychoactive drug with high affinitiy to the H1R but also to H2R^42^, was strongly inhibitory at a concentration of 30 µM (Fig. 6B). Imipramine showed equally strong inhibition of the [Ca^2+^]*i* response to ACh alone, however. It therefore appears that its inhibitory effect was primarily due to its known antimuscarinic effect^51^.

### No IgE- or IgG-receptor expression was found in P-STS cells

**Figure 7:**
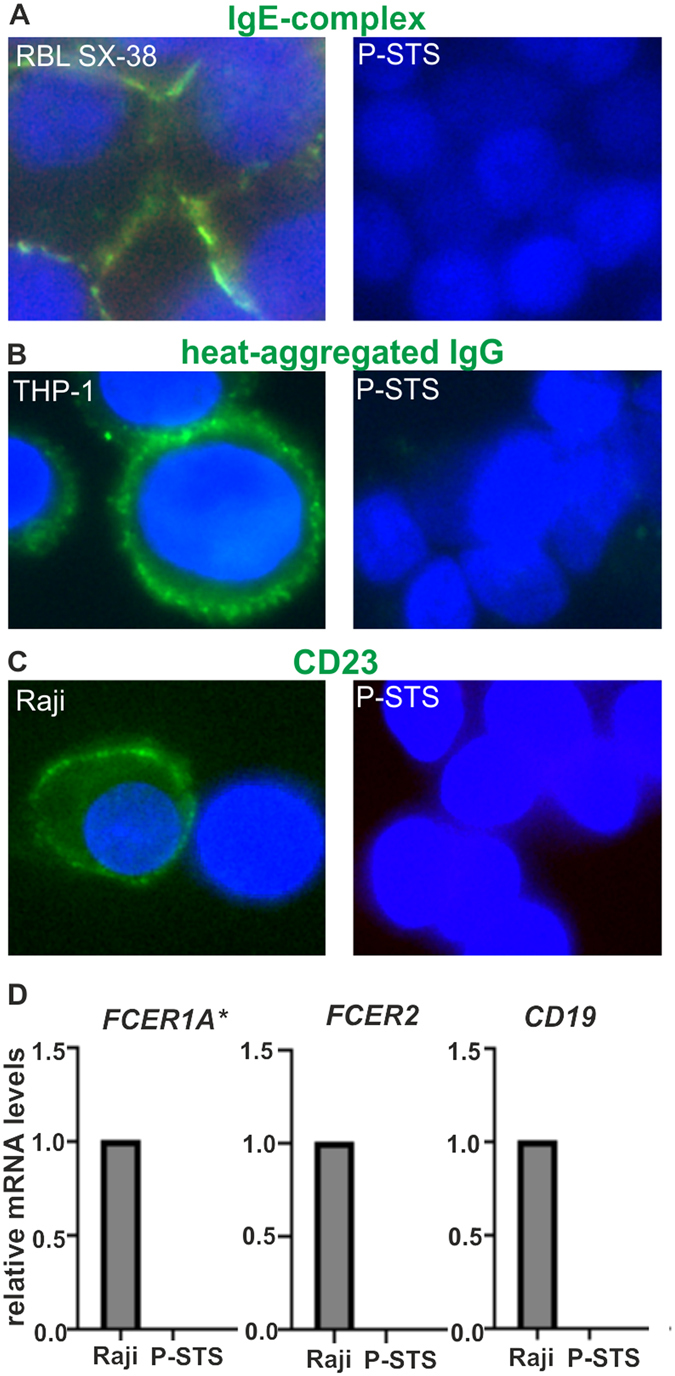
No IgG- or IgE-receptor expression was found in P-STS cells (age of culture < 6 months). (**A**) RBL SX-38 cells or Il-4 treated P-STS cells were incubated for 1 h at 37 °C with JW8/5/13 cell supernatant, washed two times with PBS and incubated for 1 h at 37 °C with NIP-BSA (100 ng ml^−1^) in pure M199/Ham’s F12 medium. The cells were then prepared for immunofluorescence staining with FITC-labeled anti-IgE. (**B**) Heat-aggregated IgG (30 µg ml^−1^) was internalized for 15 min into THP-1 cells grown with 200 nM PMA for 2 d or P-STS cells grown for 3 d with 1 µg ml^−1^ LPS, cells were fixed and prepared for immunofluorescence staining with FITC-labeled anti-IgG. (**C**) Immunofluorescence staining of Raji cells or Il-4 treated P-STS cells (both not permeabilized) with anti-CD23 (Alexa 488-labeled secondary antibody). (**D**) Expression of genes encoding IgE receptors assessed by real-time PCR analysis. Real-time PCR analysis was performed using the ΔΔCt method for relative quantification. Expression mRNA levels of the target gene were normalized to the average of the HKGs and calculated relative to the corresponding mRNA levels of Raji cells (equated to 1.0). Shown are genes encoding IgE receptors such as *FCER1A* (encoding the high-affinity receptor for IgE, alpha subunit) and *FCER2* (also known as CD23, encoding the low-affinity receptor for IgE); additionally, *CD19* is shown as the B-cell identity marker. Results are shown as mean values in one experiment ± SD and are representative of two independent experiments. *Note that *FCER1A* in Raji cells was classified as a low expressing gene in our analysis (30 < Ct < 35).

To clarify whether P-STS cells have any receptors suitable for activation of vesicle release via an immunoglobulin-mediated mechanism, complexes of NIP-BSA (see methods section) with IgE specific for this model allergen (Fig. 7A) or heat-aggregated IgG (Fig. 7B) were incubated with the cells at 37 °C and binding or internalization of immunoglobulins was monitored by immunofluorescence microscopy. While IgE complexes clearly bound to RBL SX-38 cells expressing the human high affinity IgE receptor and heat-aggegated IgG was abundantly detected associated with phorbol 12-myristate 13-acetate (PMA)-differentiated THP-1 cells, no immunoglobulin staining was seen on P-STS cells despite treatment with IL-4 for potential induction of IgE receptors or lipopolysaccharides (LPS) for potential induction of IgG receptors, as shown for other cell types^52, 53^. In agreement with this lack of IgG binding by P-STS cells, [Ca^2+^]*i* imaging after addition of medium containing non-aggregated versus heat-aggregated human IgG (30 µg ml^−1^ final concentration) showed no difference between these treatments or when compared to medium without IgG (not shown). While IL-4 was able to induce expression of the low affinity IgE receptor CD23 on THP-1 cells (positive control for IL-4 integrity, not shown) and CD23 was stained on Raji cells as a positive control, no CD23 was detected on P-STS cells cultivated with IL-4 (Fig. 7C). In line with the results of immunofluorescence microscopy and and functional assays, negative results for high and low affinity IgE receptors were also obtained on the mRNA expression level assayed by real-time PCR analysis in comparison to Raji cells (Fig. 7D).

## Discussion

The P-STS cell line was cultivated from a neuroendocrine tumor (“carcinoid”) of the terminal ileum which had already metastasized and caused flushes and bronchoconstriction in the patient^28^. In accordance with the characterization of the primary tumor and previous results from immunocytochemical staining showing 5-HT only in isolated cells^28^, only a low amount of 5-HT was detected in the cell lysate. Expression of the mRNA for TPH1, the rate-limiting enzyme for synthesis of 5-HT expressed specifically in enteroendocrine cells^1^, was confirmed. In addition, expression of synaptophysin and CgA, a protein important for vesicle biogenesis^54^, qualifies this cell line as a model for EC cells. Substance P, encoded by the *TAC1* gene and secreted by less than 20% of 5-HT-producing neuroendocrine tumors^55^, is not expressed in P-STS cells. EC cells are found in close contact with nerve endings of submucosal neurons of the enteric reflex pathways which are largely cholinergic^56, 57^. ACh secreted by enteric nerves promotes intestinal secretion and motility^56^. In rabbit duodenal^30^ and guinea pig and human colonic EC cells^7^, cholinergic agonists induce a rise in [Ca^2+^]*i*, the main physiological trigger of vesicle exocytosis. In accordance with their EC cell phenotype, the same reaction to ACh was seen in P-STS cells and - as expected^29^ – could be inhibited by simultaneous addition of SST.

The neurotransmitters isoproterenol, 5-HT or GABA did not have any statistically significant influence on [Ca^2+^]*i* or the [Ca^2+^]*i* response to ACh in the screening experiments employed. This may be due to the insensitivity of the [Ca^2+^]*i* response assay caused by large differences in response between different samples of the same type.

A rise in [Ca^2+^]*i* reaching statistical significance was induced in P-STS cells by elevation of the extracellular [Ca^2+^] from 1 mM Ca^2+^ contained in the culture medium to 6 mM in the presence of 3 mM Trp indicating the presence of functional CaSRs. Immunofluorescence microscopy showed that CaSR expression in P-STS cells was comparable in intensity and pattern to expression in THP-1-derived macrophages. However, CaSR protein was lost in almost all cells in the course of further cultivation and this was reflected in a concomitant loss of the functional response. In analogy to colon cancer cells^34^ this loss of the CaSR might be connected to the observed gradual increase in cell proliferation. In the intestine, ACh, acidic pH as well as CaSR activation enhance bicarbonate secretion by epithelial cells which is important for mucus secretion and barrier function of the gut wall^32^. 5-HT has been shown to evoke bicarbonate release from mouse duodenal mucosa via a cholinergic neuronal reflex pathway and probably also via 5-HT_4_ receptors on intestinal epithelial cells^58^, suggesting that EC cells augment responses of epithelial cells to these signals.

Unexpectedly, HA significantly enhanced the [Ca^2+^]*i* response elicited by ACh in P-STS cells. A change in [Ca^2+^]*i* is an important early event in signaling cascades elicited by HA via H1R, H3R and H4R and has also been observed for H2R^43, 59^. However, when added alone, HA caused only a slight elevation in [Ca^2+^]*i* which did not reach statistical significance. In the human intestine mast cells and neutrophils are the main sources of HA^60^. Food allergic persons have increased levels of mast cells and HA secretion in the intestine^61^. Increased secretion of 5-HT from EC cells upon antigen ingestion might contribute to symptoms like diarrhea and is thought to be proinflammatory^10^. In line with this, antigen-induced mast cell degranulation in the small intestine induced mucosal hypersecretion and muscle contractions in the antigen-sensitized guinea-pig model^62^. Mast cell activation has also been implicated in irritable bowel syndrome and symptoms in diarrhea-predominant irritable bowel syndrome tend to be alleviated by mast cell stabilizing drugs^60, 63^.

An investigation into the mechanism of HA action on P-STS cells showed that HA not only enhanced cell activation by ACh but also by the Ca^2+^ ionophor A23187, suggesting that reaching a certain level of [Ca^2+^]*i* might be enough to augment the cellular susceptibility to a second stimulus causing a rise in [Ca^2+^]*i*. Adding a tenfold higher HA concentration did not further increase the [Ca^2+^]*i* response, while the cells strongly responded to changes in final HA concentrations between 1 and 10 µM.

Our studies with HA agonists and antagonists/inverse agonists indicate the presence of functional H3Rs and H4Rs on P-STS cells, but gave no indication for the presence of H1Rs or H2Rs. Their expression might be too low for detection with inverse agonists^48^. The inhibitory effect of the selective H3R agonist methimepip on the [Ca^2+^]*i* response upon simultaneous addition of ACh or A23187 is in line with data from synaptic nerve endings where [Ca^2+^]*i* and neurotransmitter release are reduced by agonists at H3R^43, 64^.

The significant enhancing effect of the H4R agonist 4-methylhistamine on the [Ca^2+^]*i* response to ACh in our experiments indicates the presence of H4Rs on P-STS cells. Elevation of [Ca^2+^]*i* as well as degranulation and release of pro-inflammatory cytokines in response to 4-methylhistamine has previously been shown in human mast cells^65^. The apparent inhibitory effect of the H4R inverse agonist/antagonist JNJ-7777120 on the response of P-STS cells to ACh even suggests the enhancement of the cellular response to ACh by constitutive H4R activity. Constitutive activity of H4Rs has been observed in transformed cells, but up to date little if any evidence for constitutive activity in native systems has been obtained^43^. As we were not able to detect release of 5-HT or CgA from P-STS cells, we can only conclude from their [Ca^2+^]*i* response that 4-methylhistamine activates the cells less efficiently than HA when added at the same concentration, as has also been observed for eosinophil shape change^66^. Considering this, our data suggest that the enhancing effect of HA on the [Ca^2+^]*i* response to ACh might be mediated via H4R. In accordance with these putative results, H4Rs have been implicated in visceral hypersensitivity in rats^67^.

The antipsychotic betahistine^68^, and the tricyclic antidepressant imipramine are known HA receptor ligands. Betahistine is a H1R agonist^42^, while imipramine has antihistaminic as well as antimuscarinic activity^51^. Their effects on the [Ca^2+^]*i* response of P-STS cells to ACh were studied. In accordance with its lack of gastrointestinal side effects in clinical studies^68^ betahistine had no effect on the [Ca^2+^]*i* response to ACh, while imipramine inhibited this response. This is in line with the clinical observation that tricyclic antidepressants cause constipation^69^.

The facts that (i) pseudoallergic symptoms like bronchoconstriction and anaphylaxis may be associated with neuroendocrine tumors, and (ii) that the allergic mediator HA exerted a stimulatory effect on P-STS cells, prompted us to look for the presence of IgE receptors on these cells. IgE receptors on neuroendocrine cells in the gut might further contribute to an imbalance in motility and secretion in food patients with food allergy by releasing 5-HT. Crosslinking of high- as well as low-affinity IgE receptors has been implicated in raising [Ca^2+^]*i* ^52, 70^ and might therefore cause granule exocytosis from EC cells if present at the cell surface. Neither the IgE-binding α subunit of the high-affinity IgE receptor (FcεRIα) nor the low-affinity IgE receptor CD23 were detected at mRNA or protein level in P-STS cells, however. Inflammatory bowel disease patients tend to have high levels of IgG antibodies specific for luminal antigens^71^ and – although hotly debated - cancelling of foods with high serum IgG has been suggested to reduce stool frequency^72^. Not only IgE-antigen complexes, but also crosslinked IgG can induce mast cell degranulation^63^ and via the mediator HA may cause 5-HT release from EC cells. However, no indication of a direct interaction of heat-aggregated IgG with P-STS cells was found.

In this work we confirmed that the P-STS tumor cell line is a valuable model for human EC cells with expression of neuroendocrine markers, albeit low 5-HT content. Importantly, the cell line shows the rise in [Ca^2+^]*i* in response to ACh expected for EC cells. This response is significantly enhanced by simultaneous addition of HA, suggesting a participation of EC cells in symptoms of intestinal disorders associated with increased mast cell numbers like food allergy or irritable bowel syndrome. Studies with specific HA receptor ligands indicate the presence of H4Rs and of H3Rs with functional analogy to nerve cell H3Rs in this ileal EC cell line.

## Methods

### Cell culture

P-STS midgut neuroendocrine tumor cells (semi-adherent, originally isolated from a WHO III neuroendocrine tumor of the terminal ileum) were established and provided by R. Pfragner^28^. They were free of mycoplasma as judged from immunofluorescence microscopy after staining of fixed and permeabilized cells with Hoechst dye 33342. P-STS were grown in a 1:1 mixture of medium M199 and Ham’s F12 nutrient mixture supplemented with 10% heat-inactivated fetal calf serum, 100 U ml^−1^ penicillin G and 100 µg ml^−1^ streptomycin at 37 °C in a humidified atmosphere containing 5% CO_2_. Raji Burkitt’s lymphoma B cells (ATCC Catalog No. CCL-86), JW8/5/13 plasmacytoma cells (ECACC No. 87080706) secreting mouse/human chimeric IgE specific for the (4-hydroxy-3-iodo-5-nitrophenyl)acetyl (NIP) group^73^ and THP-1 monocytes (ATCC Catalog No. tib-202) were grown in RPMI 1640 medium containing the same supplements. RBL SX-38 cells^74^ (kindly provided by Jean-Pierre Kinet) were grown in DMEM medium in the presence of 250 µg ml^−1^ G418 sulfate. Treatment of THP-1 or P-STS cells with IL-4 was for 3 d at a concentration of 10 ng ml^−1^ where indicated.

### Antibodies and reagents

Mouse anti-CaSR [5C10] and donkey anti-goat Cy3-IgG were from Abcam, all other secondary fluorescent antibodies as well as FITC-conjugated goat anti-IgE and Fluo-4AM were from Life Technologies, FITC-conjugated goat anti-IgG was from Millipore, rabbit anti-synaptophysin (GTX100865) was from GeneTex, goat anti-CgA and mouse anti-CD23[BU38] were from Santa Cruz, the 5-HT ELISA kit (ADI-900-175) was from Enzo Life Sciences and the human CgA ELISA kit (ab196271) was from Abcam. A23187 Ca^2+^ ionophore, phorbol 12-myristate 13-acetate (PMA), LPS from *Escherichia coli* 055:B5, bovine serum albumin (BSA, heat shock fraction), isoproterenol, neomycin trisulfate, ACh chloride, SST, arginine monohydrochloride (Arg), HA dihydrochloride, γ-aminobutyric acid (GABA), imipramine hydrochlorid and lyophilized human IgG were from Sigma Aldrich, AC-265347 was from Santa Cruz and NPS-2143 was from Selleckchem. All HA receptor ligands (4-methylhistamine dihydrochloride, JNJ 7777120 dihydrobromide, methimepip dihydrobromide, ranitidine hydrochloride, BF2.649 (pitolisant) hydrochloride and mepyramine (pyrilamine) maleate) were from Tocris. 4-hydroxy-3-nitrophenylacetyl coupled in a ratio of 15:1 to bovine serum albumin (NIP-BSA) was from Biosearch Technologies and recombinant human IL-4 was from R&D Systems. Human IgG was heat-aggregated by incubation at 63 °C for 60 min at a concentration of 2 mg ml^−1^ in 0.9% sodium chloride and before use of the supernatant in experiments was centrifuged at 1.000× *g* for 2 min.

### EBV detection

Nucleic acid extraction was performed with 200 µl suspension of ~10^5^ cells by use of the automated NucliSens EasyMag extractor, according to the manufacturer’s instructions (Biomerieux, Marcy l’Etoile, France). EBV-specific real-time TaqMan PCR was performed with primers and probe located in the BNRF1 p143 gene (nonglycosylated membrane protein), as described by Niesters *et al*.^75^. The detection limit of real-time PCR was 200 copies ml^−1^, as determined by a commercially available, particle-counted, EBV standard (Advanced Biotechnologies Inc., Columbia, Md., USA).

### Real-time PCR analysis

Total RNA was isolated using Absolutely RNA Miniprep Kit (Stratagene by Agilent) according to the manufacturer’s protocol. 1 µg RNA was used for cDNA generation using the High Capacity cDNA Reverse Transcription Kit (Applied Biosystems by Thermo Fisher Scientific) according to the instructions of the manufacturer. Real-time PCR analysis was performed using Power SYBR Green PCR Master Mix (Applied Biosystems by Life Technologies) on ABI 7900HT instrument equipped with SDS 2.3 software (Applied Biosystems by Thermo Fisher Scientific). All primers were self-designed using the Primer Express 3.0 software (Applied Biosystems by Thermo Fisher Scientific) and validated using the Human Total RNA Master Panel (Takara, Clontech Laboratories Inc.) as described^76, 77^. Primer sequences are summarized in Table 1. For relative quantification, data were analyzed by ΔΔCT or ΔCT methods as indicated. Expression levels of target genes were normalized to the average of the selected housekeeping genes (HKGs) EEF1A1 and UBC. Ct values of genes expressed at the limit of detection were set to 40.

**Table 1:** Real-time PCR primers. Gene symbol, Gene ID and sequences of forward and reverse primers are indicated.

Gene symbol	Gene ID	Forward Primer	Reverse Primer
*CHGA′*)	1113	CTGCGCCGGGCAAGT	CATCACCTCGGTATCCCCTTT
*SYP*	6855	ACACATGCAAGGAGCTGAGAGA	CAGGTTCAGGAAGCCGAACA
*TPH1*	7166	TTCTATACCCCAGAGCCAGATACC	TGGGAGAATTGGGCAAAACT
*TAC1*	6863	GCGACCAGATCAAGGAGGAA	GGTCTCCGGGCGATTCTC
*FCER1A*	2205	CATGGAATCCCCTACTCTACTGTGT	CCTTAGGTTTCTGAGGGACTGCTA
*FCER2*	2208	AGGTGTCCAGCGGCTTTGT	AGCACTTCCGTTGGAAATTGA
*CD19*	930	TGACCCCACCAGGAGATTCTT	CACGTTCCCGTACTGGTTCTG
*EEF1A1*	1915	ATTACAGGGACATCTCAGGCTGAC	CATTCTTGGAGATACCAGCTTCAA
*UBC*	7316	ATTTGGGTCGCAGTTCTTG	TGCCTTGACATTCTCGATGGT

^′)^Gene names: chromogranin A (*CHGA*), synaptophysin (*SYP*), tryptophan hydroxylase 1 (*TPH1*), tachykinin precursor 1 (*TAC1*), Fc fragment of IgE receptor Ia (*FCER1A*), Fc fragment of IgE receptor II (*CD19*), eukaryotic translation elongation factor 1 alpha 1 (*EEF1A1*), ubiquitin C (*UBC*).

### Fluorescence microscopy

Cells grown on cover slips were fixed for 20 min with 4% paraformaldehyde in phosphate buffered saline without Ca^2+^ or Mg^2+^ (PBS), and - if not indicated otherwise - the cells were permeabilized for 5 min with 0.2% v/v Triton X-100 in PBS. Blocking and incubation with antibodies was in 5% goat serum in PBS at room temperature. Nuclei were stained with Hoechst dye 33342 in PBS. Cells were mounted in a 9:1 (v/v) mixture of glycerol and 1 M Tris-HCl (pH 8.6) containing 25% diazabicyclo(2,2,2)octane (Merck) and were viewed under a Zeiss Axioplan 2 fluorescence microscope using *Axiovision* software.

### [Ca^2+^]*i* imaging

P-STS cells were plated in 24-well plates at a density of 10^2^ to 10^3^ per well. For the experiment the medium was removed and the cells were incubated for 45 min with 3 µM Fluo-4 AM (Life Technologies, stock solution 0.4 mg ml^−1^ in DMSO) in 200 µl serum-free medium per well. Then this solution was carefully exchanged for the same volume of pure medium and the cells in 24-well plates were placed on the inverted microscope Axio Observer Z1 equipped with a high resolution AxioCam MRc 5 camera (Carl Zeiss, Jena/Göttingen, Germany) and incubated there for 30 min avoiding agitation. After focusing on a suitable group of cells, pictures were taken every 10 s (program *Axiovision*). Between the second and third picture the test substance was carefully added in 200 µl medium. For quantification the mean change in fluorescence intensity was calculated from at least 20 cells with the program *Image J*. The increase in fluorescence before addition of the test substance was extrapolated for calculation of the baseline fluorescence increase. The fluorescence increase above baseline caused by addition of the test substance was calculated from the third picture taken, e.g. from the time point 10 s after starting the addition of the test substance.

### Statistical analysis of [Ca^2+^]*i* responses

It was obvious that increases in fluorescence calculated from the [Ca^2+^]*i* imaging experiments did not display a normal distribution, especially when using strong agonists (an example is given in Fig. 5A right panel). Additionally, the strength of reaction to the same agonist varied between experiments, possibly due to differences in cell density influencing mutual inhibition or activation between adjacent cells^78^. Statistical significance of the differences of mean values between 2 samples of interest was calculated by the non-parametric Mann-Whitney *U*-test using IBM SPSS Statistics for Windows version 24 (IBM Corporation, USA). The frequency distributions obtained with strong agonists appeared strongly right skewed or bimodal and as very high values are not adequately considered by rank tests, the Mann-Whitney *U*-test might for these cases underestimate statistical significance. For some of the data statistical significance of the difference of mean values was also calculated from ranked data with the unpaired two-tailed *t*-test assuming unequal variances^79^. The slightly lower *p*-values obtained with this procedure are given in the figure legends. All samples were considered independent. Experiments were initially conducted as screening experiments with different sample numbers per group. To increase statistical reliability and strength, in all further experiments all treatments were conducted in parallel resulting in equal sample numbers in each treatment group.

### 5-HT quantification in cell lysate and supernatant

P-STS cells were plated at a density of 10^6^ cells per well in a 24-well plate. The cells were washed with PBS, detached with trypsin/EDTA (removed immediately) at 37 °C and resuspended in 5% w/v BSA in serum-free medium. An aliquot was lysed by three times freezing and thawing followed by 3 min of treatment in a sonication bath and the 5-HT content of the supernatant after high-speed centrifugation was determined by ELISA following the instructions in the kit. Per well of the 96-well ELISA plate 90 µl of sample (containing 5% BSA) were added to 10 µl of assay buffer. 5-HT standards were added to the same matrix.
